# DEVELOPING A FRAILTY INDEX USING THE ELECTRONIC HEALTH RECORDS TO EXPLORE THE POLYPHARMACY AND FRAILTY RELATIONSHIP

**DOI:** 10.1093/geroni/igae098.2754

**Published:** 2024-12-31

**Authors:** Reham Yasin, Deborah Lekan, Laurie Kennedy-Malone, Thomas McCoy, Anna Boone, Cindy Bacon

**Affiliations:** King Saud bin Abdulaziz University for Health Sciences- College of Nursing Jeddah, Jeddah, Saudi Arabia; University of North Carolina Greensboro, Greensboro, North Carolina, United States; University of North Carolina Greensboro, Greensboro, North Carolina, United States; University of North Carolina Greensboro, Greensboro, North Carolina, United States; Rockingham Gastroenterology, Reidsville, North Carolina, United States; University of North Carolina Greensboro, Greensboro, North Carolina, United States

## Abstract

The association between polypharmacy and frailty among hospitalized adults was explored using a secondary data analysis of the EHR in adults aged 50 years or older (N=46,645) hospitalized in medical-surgical units between 2013 and 2017. A Frailty Index (FI) was created based on the accumulation of a deficit theoretical framework (Mitnitski et al., 2001). The FI was constructed following the guidance provided by Searle et al. (2008) and Theou et al. (2023). This study’s FI was unique because it included indicators from nursing flowsheets, ICD-10 codes, laboratory blood biomarkers, as well as other frailty indicators (35 deficits in this study). The indicators were coded as “1” for present and “0” for absent. FI-35 was calculated by dividing the number of indices in an individual by the total number of indices measured, with frailty defined by a cut-off point of ≥ 0.25. In the study (25,961), 42.0% were identified as frail. A significant association was found between polypharmacy and frailty. The study showed 54.5% of individuals with polypharmacy classified as frail, compared to 31.4% in the non-polypharmacy group (χ²=1283.886; p< 0.001). The odds of frailty were 53.1 % higher for those in the polypharmacy groups relative to non-polypharmacy groups, adjusting for age and Elixhauser comorbidity index score (AOR=1.531, 95% CI=[1.437, 1.631], p< 0.001). When recognizing frailty in hospitalized patients with known polypharmacy, consideration should be made for medication reconciliation and medication deprescribing before discharge.

